# Adaptive group testing strategy for infectious diseases using social contact graph partitions

**DOI:** 10.1038/s41598-023-39326-9

**Published:** 2023-07-26

**Authors:** Jingyi Zhang, Lenwood S. Heath

**Affiliations:** Department of Computer Science, Virginia Tech, Blacksburg, VA 24060 USA

**Keywords:** Computational models, Infectious diseases

## Abstract

Mass testing is essential for identifying infected individuals during an epidemic and allowing healthy individuals to return to normal social activities. However, testing capacity is often insufficient to meet global health needs, especially during newly emerging epidemics. Dorfman’s method, a classic group testing technique, helps reduce the number of tests required by pooling the samples of multiple individuals into a single sample for analysis. Dorfman’s method does not consider the time dynamics or limits on testing capacity involved in infection detection, and it assumes that individuals are infected independently, ignoring community correlations. To address these limitations, we present an adaptive group testing (AGT) strategy based on graph partitioning, which divides a physical contact network into subgraphs (groups of individuals) and assigns testing priorities based on the social contact characteristics of each subgraph. Our AGT aims to maximize the number of infected individuals detected and minimize the number of tests required. After each testing round (perhaps on a daily basis), the testing priority is increased for each neighboring group of known infected individuals. We also present an enhanced infectious disease transmission model that simulates the dynamic spread of a pathogen and evaluate our AGT strategy using the simulation results. When applied to 13 social contact networks, AGT demonstrates significant performance improvements compared to Dorfman’s method and its variations. Our AGT strategy requires fewer tests overall, reduces disease spread, and retains robustness under changes in group size, testing capacity, and other parameters. Testing plays a crucial role in containing and mitigating pandemics by identifying infected individuals and helping to prevent further transmission in families and communities. By identifying infected individuals and helping to prevent further transmission in families and communities, our AGT strategy can have significant implications for public health, providing guidance for policymakers trying to balance economic activity with the need to manage the spread of infection.

## Introduction

During the coronavirus (COVID-19) pandemic, governments worldwide reacted by imposing lockdowns, many companies switched to remote work, and schools provided education online. These policies incur considerable economic costs. Therefore, finding a proper way to lift lockdowns and bring people back to normal life is critical. Indeed, lockdown policies are complex political, health, social, and economic issues. A significant risk exists that, once the pandemic slows down or appears to be under control and lockdown measures are lifted, new waves of COVID-19 will appear.

This suggests that, during an epidemic, it is crucial to test individuals efficiently and effectively to control the epidemic and reduce the consequences. Testing is important for early diagnosis and treatment, prevention and control of the epidemic, and resumption of work and production. Especially, mass testing, also known as large-scale testing, enables rapid detection of infections and helps break chains of transmission in the community. Reliable mass testing can detect infected individuals early, including asymptomatic individuals, so that early isolation and treatment measures can be taken to avoid secondary transmission and reduce the risk of developing severe symptoms later. It will also allow those who test negative to return to work without risking another wave of contagion. Mass testing was adopted during the prevention and control of the COVID-19 outbreak in Beijing’s Xinfadi Market and in Dalian, Liaoning Province, and successfully reduced the number of severe cases^1^. Countries including Slovenia, Georgia, the United Kingdom, Germany, and France also launched mass testing or community-scale pilot mass testing to monitor the infection prevalence and to control the COVID-19 outbreak^2^.

Nevertheless, to make mass testing effective, it is important to have efficient testing strategies and methods, a reasonable turnaround time, skilled testing personnel, and a reliable supply of testing materials. For COVID-19 testing, the gold standard is Real-Time Polymerase Chain Reaction (RT-PCR)^3^, which involves a chemical reaction that produces fluorescent light if viral DNA is present in a sample. RT-PCR has two steps, first taking samples from individuals, then amplifying parts of the virus DNA known as markers through a PCR machine. The first step is straightforward but requires trained healthcare professionals to administer. The second is a bottleneck, limiting the testing capacities, as the chemical lab work requires time and reagents. Scaling up the capacity of RT-PCR testing for the SARS-CoV-2 virus responsible for the COVID-19 requires time and money. However, to contain an epidemic, mass screening is more effective the earlier it is done. In the current practice, daily testing^4–7^ is considered to be an efficient way to monitor the spread of infection and quickly identify any new cases. This requires a testing capacity that can handle the daily volume of samples and provide results in a timely manner. Moreover, a surge in testing demand may overwhelm the workforce and create a shortage of testing consumables. And the surge may increase the testing turnaround time. The rapid turnaround of tests is crucial to ensure the efficiency and effectiveness of case isolation and timely treatment. When susceptible individuals are waiting for testing or the results of testing, undetected infectious individuals may spread the virus, leading to secondary transmission and making the testing even less effective. Each of these will limit the rollout of mass testing.

Therefore, to improve testing efficiency, experts have recommended group testing. By pooling samples from a group of individuals in one tube and testing all of them at once, this method can decrease the total number of tests required and the testing turnaround time. Harvard economist Robert Dorfman^8^ first proposed group testing in the 1940s to detect how many World War II soldiers were carrying syphilis. Often it has also been employed to screen how many people in asymptomatic populations carried, for example, chlamydia and gonorrhea bacteria. The Red Cross also uses group testing to screen blood donors for hepatitis B and HIV. However, Dorfman’s method still has some drawbacks. One is the lack of a uniform standard for the upper limit of the number of samples in a single group, which can result in the dilution of the pathogen and false negative test results. Additionally, group testing requires a two-round testing process. If the first round of testing identifies those groups that have at least one positive, the second round then tests each individual separately to confirm their infection status. If the turnaround time for each round of testing is long, such as with RT-PCR, it can significantly extend the overall testing time and defeat the goal of mass testing.

Traditional group testing methods such as Dorfman’s usually assign individuals to the group at random^4,8–18^. Yet, this ignores the potential correlation of infection risk among individuals. Diseases such as COVID-19 can spread from person to person through droplets, primarily among close contacts. Individuals who share similar social activities in their daily lives may be at similar risk of developing the disease. Infected individuals are also more likely to spread the disease to those with whom they are socially connected. Group testing research has not explored such functionality with social contact networks. Consequently, there is an opportunity to develop an efficient group testing method that can effectively reduce the overall number of tests required to screen the entire population and reduce the number of new infections caused by a secondary transmission during the testing period.

To address the challenges above, we propose an adaptive group testing (AGT) strategy, based on a social contact network, with fixed, limited testing capacity at each time instance, where complete testing of the entire population is not possible for economic or logistic reasons. The strategy consists of: (1) a near balanced graph partition of the social contact network according to the testing capacity and (2) an adaptive testing order assignment mechanism based on point prevalence (as defined in “Adaptive group testing strategy” Section) and disease transmission trajectory. In addition, we present an agent-based compartment model to capture the spreading characteristics of SARS-CoV-2, incorporating heterogeneous infectiousness. We use this model to simulate the disease transmission process on social networks and use the simulation results to validate the effectiveness of our group testing strategy. Also, some small real world networks are studied.

## Prior literature

One classic group testing method is the Dorfman two-stage group testing method^8^. The collected independent samples are first organized into groups. A portion of each sample is then extracted and combined into a single tube to create a group sample for testing. Instead of testing each individual sample, a direct test is performed on the group sample. A negative test result for the group sample indicates the absence of infection in all individuals within the group, whereas a positive test result indicates that at least one individual in the group is infected and that all individuals must be tested to identify infected individuals. Dorfman’s method is the first non-overlapped method, where individuals being tested are not repeated or duplicated across different groups, as opposed to an overlapping method^9,10,17,18^ where the same individuals are tested multiple times in different groups. The work in^19^ performed Monte Carlo simulation analyzes with varying prevalence and group sizes, suggesting routine group testing would substantially reduce the number of tests required to screen a population. Studies showed that adequate use of group testing strategies could contain the spread of a disease^20,21^ and save between 85 and 95% of testing resources depending on the precise situation^16^.

Most group testing methods employ RT-PCR to perform the testing. However, the cost of a single test is high, and the testing process requires several hours. Especially, due to the two-stage design, performing the second round of testing requires the results of the first round, which leads to a longer turnaround time. To further reduce the cost and speed up the testing, many new testing technologies are introduced to facilitate group testing. Various COVID-19 testing technologies differ significantly in specificity, sensitivity, turnaround time, costs, and the types of samples the tests use. More and more antigen and antibody tests, such as the IgG-IgM-coated antibody detection and the rapid lateral flow coronavirus (COVID-19) tests, have been approved by the FDA and are being deployed^22,23^. Although the total number of tests increases, rapid testing is not limited by the testing capacity, allowing more people to test and obtain real-time feedback simultaneously. Moreover, these new testing methods make the cost of a single test lower and are more economical^7^. However, due to low sensitivity, false negatives can allow more secondary transmission and may not effectively contain the spread of the virus^6^. While RT-PCR is the current gold standard testing method, individual RT-PCR testing is still required after these rapid screens to confirm the diagnosis. As a result, the healthcare system is under even greater strain.

In the context of the pandemic, a number of variations of group testing^9,10,13–18^ have been proposed, building on Dorfman’s method. Combinatorial group testing methods place the samples into overlapping pools, such that every single sample appears in a unique combination of pools^10,13,15,17^. If all the samples in the unique set of pools return a positive result, the infected individual can be identified without requiring an additional round of testing, reducing the overall tests needed. However, these combinatorial group testing strategies generally assume that a complete screen of the entire population is possible. While the supply and the resources may be limited in the early stages of an emerging disease outbreak or in less developed areas, the testing capacity is not sufficient to test all of a population at once. Undetected infected individuals may infect susceptible individuals who have already obtained negative results. Therefore, group testing methods without considering the order and testing capacity can lead to more secondary transmissions, further weakening their effectiveness.

To meet the requirement of the testing capacity, a number of researchers^5,11,12^ introduced adaptive group testing methods. Based on the results of the previous week’s testing results, such models can determine the current optimal group size or the optimal number of group tests by estimating the community prevalence. Yet, these methods assume that disease transmission in the population is homogeneous, namely, infected individuals can infect any susceptible individuals. Said another way, the probability of infection is the same for close family members and strangers with no social contact. As a result, simulation results differ from reality.

While many Dorfman-based methods assume that groups are chosen at random, recent studies have identified the importance of grouping family members or other close contacts together^24–26^. However, as people tend to maintain a much more complex contact network in the real world, effective grouping cannot be done solely on a social characteristic. Recent research^4,7^ has introduced a multilayer contact network for disease transmission simulation^27^ and incorporates the individual’s health history questionnaire and demographic information to estimate the risk level of each individual by introducing a regression model to assign testing subgroups. The more information available, the more efficient the testing strategy can be. Nevertheless, these models fail to address the dynamics during the testing time frame. Therefore, designing effective testing groups over time remains a challenge.

## Problem statement

Two general computational problems are involved in our strategy: social contact network partitioning and testing group priority adjustment.

### Social contact network partition

We are given an undirected and unweighted social contact network $G = (V, E)$, where individuals constitute the set of vertices *V*, the set of edges *E* records the social contacts between individuals in pairs, and the population size is $\left| V \right| = N$. We assume that individuals in the same subgraph have similar social contact patterns and, therefore, share related infection correlations. The maximum number of individuals allowed to pool together for testing using the given testing method (think RT-PCR) is *k*. In practice, *k* for PCR testing can range from five to ten samples per pool^18^ up to several hundred samples per pool^13^, depending on the specific testing protocol and the prevalence of the disease.

Dividing individuals into more homogeneous groups based on their social interactions can help identify subgroups that are more likely to have been exposed to a shared transmission event. For example, individuals who are part of the same subgraph are more likely to have common contacts and be exposed to the same infectious agent. With the help of proper network partition, group testing can be conducted more efficiently, allowing for greater identification of infected individuals and limiting the spread of infectious diseases. The Network Partition problem is to partition *V* into subsets $V_1,V_2,\ldots , V_m$ such that each $V_i$ has $\le k$ elements, each $V_i$ induces a subgraph $G_i=(V_i,E_i)$, and the sum $\sum _{i=1}^m |E_i|$ is maximized.

### Testing group priority adjustment

As discussed in the “Prior Literature” Section, limited supplies and resources make it difficult to test the entire population for an emerging disease outbreak, yet it is critical to identify infected individuals to control the spread of the disease. Undetected infected individuals can continue to spread the disease to susceptible individuals, even after receiving negative test results. To address this challenge, testing strategies that take into account the dynamic changing infection status of each individual over time can be valuable. The second computational problem, Priority Adjustment, seeks to determine who to test at each time *t*.

For the testing scenario, we assume we have a partition of *V* as described in  “Social Contact Network Partition” Section. For each subgraph $G_i=(V_i,E_i)$, we consider the set $V_i$ of individuals as a potential group for testing at time *t*. And due to the limited testing capacity, not every potential testing group can be tested at each time point. Instead, a subset of the potential testing groups is considered for testing at each time. For a testing group $V_i$, the group test pools the samples from $V_i$ and records the testing result as $X_{i,t}$.

In the two-stage testing design (as shown in Fig. 2), based on Dorfman’s method^8^, $X_{i,t}$ refers to the number of individuals who test positive in the second round of testing at time *t*, given that the first-round testing result is positive. In the absence of a positive first-round test result, $X_{i,t}$ is 0. Thus, the total number of infected individuals *Y* detected over the course of *T* testing periods is given by:

$$\begin{aligned} Y= & {} \sum _{t = 0}^{T}\sum _{i = 0}^{m}X_{i,t} \end{aligned}$$

Given that the test capacity is insufficient to test all groups at once, we assign a testing order to each testing group based on the previous day’s test results, the point prevalence, and the topological connectivity of each group. A higher priority will be given to groups with positive test results from neighbors (as shown in Fig. 2).

Let the maximum number of tests per day be *B*, $Q_{i,t}$ denotes the number of tests performed on testing group $G_i$ at time *t*, where

$$\begin{aligned} Q_{i,t}= & {} {\left\{ \begin{array}{ll} 1, &{} X_{i,t} = 0 \\ 1+\left| V_i \right| , &{} X_{i,t} \geqslant 1 \\ \end{array}\right. } \end{aligned}$$

We will only test the top $z_t$ groups, where $z_t$ is the maximum number of groups can be tested given the testing capacity *B*. Then at any discrete time *t*, we have a restriction that $\sum _{i = 0}^{z_t}Q_{i,t} \leqslant B$ for some constant bound *B*.

Therefore, we consider the testing group priority adjustment at each time step *t* as follows: given an undirected and unweighted social contact network *G*, group test result $X_{i, t-1}$, and testing capacity *B*, the problem is to find possible $z_t$ groups that satisfy $\sum _{i = 0}^{m}Q_{i,t} \leqslant B$ and maximize $\sum _{i = 0}^{m}X_{i,t}$.

The notations and parameters used in this work are summarized in Table 1 for clarity and convenience.

## Data sets

In this study, 13 contact networks with diverse degree distributions are considered inputs for our strategy. All networks are unweighted and undirected.

We generate six random graphs to illustrate the social contact network using the following generation models in a wide range of parameter settings. Table 2 summarises generated networks along with their generation model, the number of nodes *N*, the average degree $\left\langle k \right\rangle$, and the diameter *d*. All networks are set to have 1,000 individuals, and the expected average degree is set to $\left\langle k \right\rangle = 6$. However, the network diameter *d* varies from 5 to 11 due to the nature of different generation models. We consider these synthetic networks are equivalent but with different network structures.

Moreover, we use seven data sets collected by the SocioPatterns collaboration from various real social contexts: a workplace, with data collected in two different years (InVS13, InVS15)^28^, a hospital (LH10)^29^, a primary school (LyonSchool)^30^, a scientific conference (SFHH)^31^, a high school (Thiers13)^32^, and a village in rural Malawi(Malawi)^33^. Table 3 lists the seven real social contact networks as well as the data collated location, year, the number of participants *N*, the total duration of the data collection *T*, the average degree $\left\langle k \right\rangle$ and the diameter *d*.

## Methods

**Figure 1:**
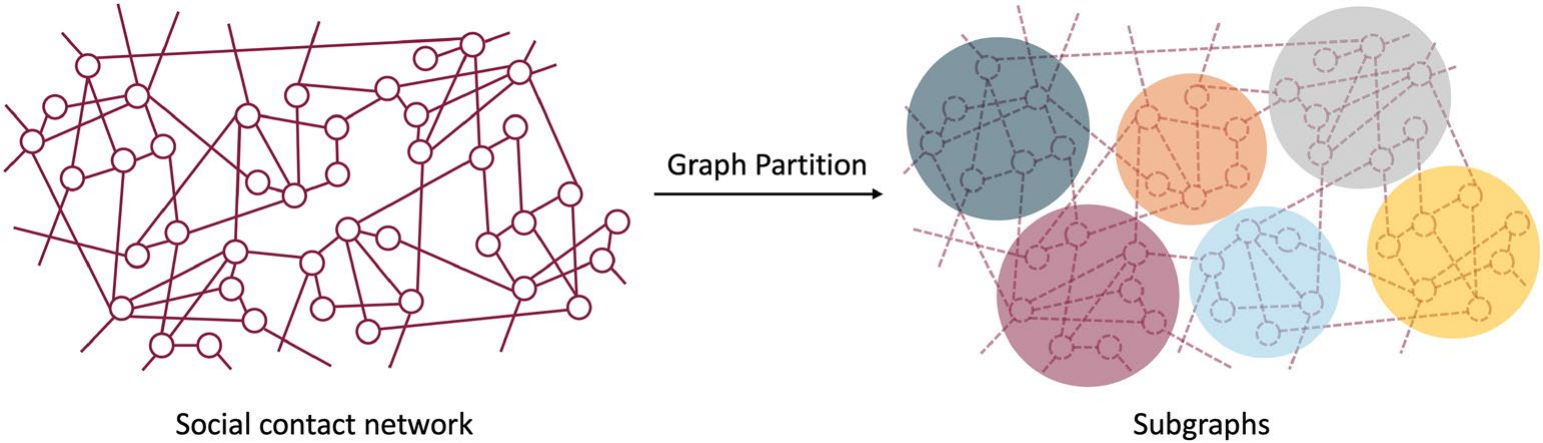
A demonstration of social contact network partition.

To maximize the underlying group correlations, we start this work by partitioning the social contact network into a number of near-balanced subgraphs to form testing groups as shown in Fig. 1. Our proposed testing strategy then takes the partition result as the input and puts groups prioritized by the strategy into the 2-stage testing. We also introduced an enhanced infectious disease transmission model to simulate the virus’s dynamic spread on the given contact network and to evaluate the performance of the testing strategy.

### Graph partition

We select *k* as the upper bound on the number of vertices in each subgraph and define $M_G$ as the partition score function to calculate the maximum number of edges in the combined subgraph without exceeding size *k*. The purpose of the $M_G(G_i, G_j, k)$ partition score function is to assess the quality of merging two subgraphs, $G_i$ and $G_j$, while taking into account the upper limit on the number of vertices, denoted by *k*. If the total number of vertices (individuals) in the union of $G_i$ and $G_j$ does not exceed the size limit, the function returns the total number of edges in the union. This quantifies the potential connections within the combined subgraphs. However, if the total number of vertices in the union of $G_i$ and $G_j$ exceeds the size limit, the function returns -1, indicating that merging these two subgraphs is not feasible within the given size constraint. Specifically, $M_G$ is defined as follows:

$$\begin{aligned} M_G(G_i, G_j, k)= & {} {\left\{ \begin{array}{ll} \mid E_i \cup E_j \mid + \sum _{u \in V_i, v \in V_j} \mid (u,v) \mid &{} \mid V_i \cup V_j\mid \le k;\\ -1 &{} \textrm{otherwise}.\\ \end{array}\right. } \end{aligned}$$

We use Algorithm $\textsc {Max-Intra-Group-Edges-Partition}(G, k)$ in Fig. 3 to decompose the graph into subgraphs. The algorithm follows a greedy approach that starts with singleton groups and merges smaller groups by maximizing $M_G$. By maximizing the intra-group edges, we find that individuals within each subgraph $G_i = (V_i, E_i)$ share correlated infection probabilities induced by the social contact network. Once the group partition is performed, the composition of the groups remains constant throughout all future testing steps.

### Adaptive group testing strategy

In our idealized testing scenario, the tests are perfect, and test results are available immediately. As the disease spreads within the social contact network *G*, we apply the testing strategy, where a limited number of tests are performed to detect a number of infections in our settings. Since the purpose of detection varies at different stages of the disease outbreak, we introduce two strategy modes: Tracing mode and Screening mode.

- Tracing mode:
  - At a low prevalence time, we focus more on containing the secondary infections and reducing the regional outbreak size. Early detection of infected individuals is beneficial in achieving these goals.
  - In Tracing mode, higher test priority is given to the neighbors of the infected individual, as they are more likely to have been in contact and may be at higher risk of infection.
- Screening mode:
  - When prevalence is high, whole population screening will quickly help identify the most infected individuals.
  - In Screening mode, priority is given to breadth-first testing, which aims to cover a wide range of individuals to efficiently identify the most infections.

The mode switch is determined by the point prevalence, $\rho _t$, which represents the ratio of infected individuals tested to the total number of individuals tested at current time *t*. It is defined as

$$\begin{aligned} \rho _t= & {} \sum _{i = 0}^{z_t}\frac{X_{i,t}}{Q_{i,t}}, \end{aligned}$$

where $X_{i,t}$ represents the number of individuals who test positive in group *i* at time *t*, and $Q_{i,t}$ represents the number of tests performed on testing group $G_i$ at time *t*.

We use Algorithm $\textsc {Testing-Priority-Adjustment}(\rho _{t-1}, \beta , previous\_results)$ in Fig. 4 to adjust the testing priority of groups adaptively based on the point prevalence $\rho _{t-1}$ and the threshold $\beta$. The value of $previous\_results$ encompasses the historical data of the testing outcomes from time step $t_0$ to $t-1$, including the number of tests and the corresponding test results. This process ensures that the appropriate mode (Tracing or Screening) is selected and the groups are prioritized accordingly. At each time step, the function takes the point prevalence, $\rho _{t-1}$, calculated from the previous time step, and compares it with a predefined threshold. The threshold represents the value at which the mode switch occurs. The algorithm then returns the sorted groups, reflecting the adjusted testing priority based on the chosen mode.

Our objective is to maximize the total number of infected individuals *Y* detected by our testing strategy, as well as minimizing the total number of tests $Q_{i,t}$, as outlined in “Testing Group Priority Adjustment” Section.

We describe the overall AGT (Fig. 5) as follows:

1. Partition the social contact network into *m* subgraphs to form testing groups.
2. Perform group testing within the constraint of the maximum number of tests.
3. Estimate the current point prevalence using the test results to determine the strategy mode (Tracing or Screening).
4. Adjust the testing priority of the corresponding groups based on the point prevalence $\rho _{t-1}$.
5. Repeat steps 2–4 iteratively until the given testing time period ends.

### Agent based infectious disease transmission model

As the traditional SEIR differential equation model is a population-scale model, it considers how many individuals flow from one state to another. To take spatial and human behavior into account, here we employ an agent-based model (ABM). ABM can simulate many individual agents in the population. Individuals can be heterogeneous and have multiple attributes. Each individual can interact with others along the contact network and is updated in random order.

We describe the infectious disease transmission process using an enhanced agent-based SEIR model, with heterogeneous infectious probabilities to incorporate the characteristics of SARS-CoV-2 infection, as shown in Fig. 6. In particular, each individual has a random infectious probability drawn from a pre-defined exponential distribution $X \sim \textrm{Exp}(\lambda )$. The expected infectious probability is given by $\frac{1}{\lambda }$. Detailed information about the model is as follows.

At any discrete time *t* between 0 and *T*, each individual $V_i \in V$ can be in one of the following states: *S* (Susceptible), *E* (Exposed), *I* (Infectious), *R* (Removed). Given a contact network *G*, new infections only occur during social contact between infected and susceptible individuals if they have an edge in *G*. An individual *i* moves from *S* to *E* with probability $\lambda _j$ if one of *i*’s neighbors $j \in$*I*. The infected individual’s infection probability $\lambda _j$ is a random number drawn from an exponential distribution. After a set incubation period, the *Exposed* individual becomes infectious and will move to the *Infected* state. Each infected individual draws a random recovery period from a normal distribution with mean $\mu$ and standard derivation $\sigma$. After the recovery period, the infected agent moves to the *R* (Removed) state. Individuals that have already moved into *R* will not be reinfected.

Table 4 lists all simulation parameters.

To start the simulation, all individuals are initially in the susceptible state. We then introduce the external infection as the start by randomly selecting $N_0$ individuals from the population to become infected at time step 0.

### Evaluation

We evaluate our proposed strategy and baselines with our enhanced SEIR model on both synthetic and real social contact networks.

There are three baseline strategies:

- *Strategy 1* Individual testing (IT) randomly draws *B* individuals from the population without exceeding the testing capacity, and tests individually.
- *Strategy 2* Random Group testing (RGT) applies randomly selected groups as described in the original Dorfman’s method^8^. The group size is *k* and the number of tests performed at each time step is $\lfloor \frac{B}{k+1}\rfloor$.
- *Strategy 3* Graph partition based group testing with random testing order (G-RGT) is a simplified version of our testing strategy. It only focuses on the underlying information collected from the social contact network. This strategy first divides the social contact network into *m* groups, then randomly selects $\lfloor \frac{B}{k+1}\rfloor$ to perform the two-stage testing.

We run the simulation for 75 time steps on the real contact networks and 150 on the synthetic contact networks, where each time step corresponds to a day. The group size is 10 and the testing capacity is 150 for synthetic networks, though we include a study varying the group size and the testing capacity. The threshold $\beta$ for switching the strategy mode is 0.02. As the total population of the real contact network varies, we tentatively set 15% population as the testing capacity. Note that the present analysis assumes perfect testing and complete quarantine compliance, with the latter represented by a quarantine rate of 100%. Upon quarantine, the edges associated with the infected individuals will be removed from the graph, thus interrupting any further transmission of the infectious agent. These assumptions are made for the purpose of exploring the optimal performance that can be achieved under idealized conditions. We compare these testing strategies in terms of the total number of tests, the maximum outbreak size, the maximum number of secondary transmissions, and the number of uninfected individuals by the end of the simulation period. We report the average of results over 100 simulations.

## Results

We simulate AGT on six synthetic contact networks and seven real networks and compare it with three baseline strategies (IT, RGT and G-RGT). Also, we explore the testing performance of AGT at different population scales, group sizes, and testing capacities.

Table 5 and Fig. 7 show the performance of testing strategies on synthetic contact networks with population 1000. IT achieves the poorest results in the total number of tests. In contrast to individual testing, all three group testing strategies achieve significant reductions in the number of tests and lead to savings of up to 90.56%. As the groups in RGT are randomly pooled from the whole population, it provides the most comprehensive coverage. Even though RGT is able to reduce the overall number of tests compared to individual testing, the difference in the resulting secondary transmission and outbreaks is not significant. The performance of the G-RGT (strategy 3) yields the worst among group testing strategies. Although G-RGT detects the highest number of positive infections, the corresponding outbreak size and the total number of tests are also significantly higher than other strategies. Surprisingly, IT gives the smallest maximum outbreak size and fewer secondary transmissions on the SW network. Other than that, AGT consistently outperforms other testing strategies. Since the network structure is heterogeneous, our AGT can save about 87.90–90.56% of tests compared to the individual testing method. AGT successfully reduces the maximum outbreak size by about 3.99–46.66% as compared to other strategies (except for the SW model), which helps reduce the burden on the public healthcare system during outbreaks. Moreover, with the help of the tracing mode in AGT, the number of secondary transmissions is lowered by approximately 1.97–27.49%.

Figure 8 shows simulation results for the number of infected (left) and uninfected (right) individuals per day on the Chung-Lu (CL) network with all four testing strategies. Results for the remaining network generation models follow a similar pattern and are omitted here. As can be seen in the left plot, AGT successfully both lowers and postpones the outbreak peak, with no significant spike throughout the simulation. Due to the timely detection of infected individuals, no large regional outbreaks result from secondary transmission. Thus, as shown on the right, our AGT protects a greater number of susceptible individuals. In contrast, G-RGT displays strong instability. There are multiple repeated rallies in the early period of the outbreak. It only gradually declines after reaching the peak of the outbreak. IT and RGT performed roughly similarly, but RGT is more volatile and declines more slowly after reaching the outbreak peak. Thus, in the right part we can see that RGT leads to fewer uninfected individuals than IT.

We also conducted experiments to compare the performance of AGT with baseline strategies under varying transmission settings. Taking the CL network as an example, Fig. 9 presents the simulation results, showcasing the comparison of AGT with the baseline strategies. The results demonstrate the superiority of AGT across different transmission settings. AGT consistently outperformed the baselines by achieving a higher percentage of uninfected individuals and requiring a lower total number of tests. This highlights the effectiveness of AGT in containing the disease transmission while optimizing testing resources. The advantages of AGT become even more significant as the disease transmission capability increases, providing protection to a greater number of people. Although IT achieved a slightly lower outbreak size at very high transmission setting ($\lambda$ = 4), it consumed 576.25% more resources compared to AGT. Moreover, AGT exhibited stable performance in controlling secondary transmissions, ensuring effective containment of the spread of disease. Notably, the outbreak size could be effectively controlled to the initial outbreak size (5) within a specific range of transmission settings ($\lambda$ range of 6–14). These findings underscore the significant advantages of AGT over other baseline strategies, emphasizing its potential of adaptability in diverse transmission scenarios, ensuring robust and efficient containment measures.

Furthermore, we simulate various testing capacities ranging from 5 to 25% of the whole population to investigate how testing capacity affects strategy performance. Figure 10 shows the simulation results of AGT on six synthetic networks at different test capacities. In general, the greater the testing capacity, the greater the number of susceptible individuals that will be protected from infection, the smaller the outbreak size, and the fewer the number of secondary transmissions. The spread of the disease is not effectively contained on any network model when the testing capacity is 5% of the whole population since the limited testing resources are insufficient. Especially on the BA model, the outbreak size reached 15.13% of the total population. When the testing capacity rises to 10%, it only performs well on the SW model but still does not effectively protect susceptible individuals on other network models. There are no significant differences on each evaluation measurement after the test capacity reached more than 20%: approximately 95.89–97.96% of susceptible individuals are protected from infection, the outbreak size is about 5–5.74 (slightly larger than the initial outbreak size $N_0$), and secondary transmissions ranged from 5.30 to 11.99.

Additionally, we compared the performance of AGT with different group sizes, as shown in the Fig. 11. As the group size increases, the strategy’s performance does not keep improving. The optimal group size varies slightly across network models instead. When the group size is 20, AGT performs better on some network generation models, such as CL, ER, and SBM, which allowed maximum protection of the susceptible population but required fewer tests. On the SW and Waxman network models, a group size of 15 resulted in the lowest number of infections and the smallest outbreak size.

We also investigate how the population scale affects the performance of the testing strategy. We simulate four population scales ranging from 500 to 2000, and set the initial outbreak size as 1% of each population and the test capacity as 15% of the corresponding population. All the other parameters remain unchanged. As can be seen in Fig. 13, the performance of IT gradually becomes more deficient as the total population climbs, while the performance of group testing strategies gradually improves. Our proposed strategy consistently protects the most susceptible individuals from infection when the population scale increases, regardless of the network structure. Moreover, AGT is always optimal in terms of saving testing resources and reducing secondary redirection throughout all simulations. It saves 87.00–90.96% of testing resources compared to IT and achieves a 7.49–20.19% reduction compared to RGT.

Since we noticed that the total population of each real data sets is limited, but the average degree is relatively high, the spread of the disease may be greater than the ones we simulated on the synthetic network. Here we further develop a variation (AGT_var) of our proposed strategy, which gives higher priority to the “Tracing mode” when the estimated prevalence is high. Table 6 and Fig. 12 show the performance of testing strategies on seven real contact networks. Generally, group testing strategies (Strategy 2, 3, 4 and its variation) perform better than IT (Strategy 1). Group testing strategies can save 76.08–88.41% of testing resources, but the difference among different group testing strategies is insignificant. It is worth noting that our original strategy is not effective in reducing the outbreak size on high-density real networks. The variant for high-density networks significantly reduces the outbreak size only on the SFHH network and does not differ much from the random method on other data sets.

## Discussion

Our study shows that group testing is typically more efficient than individual testing. AGT saves testing resources up to 90.96% across simulated scenarios with limited testing capacity. AGT effectively identifies the infected person early in the spread of the infection. This demonstrates that timely and effective public health interventions are critical in the early stage of an infectious disease outbreak. It can lessen pressure on the public health system by reducing the size of the outbreak and delaying the arrival of the peak to give more time for a series of public health emergency responses. However, these results are obtained under several assumptions, such as the disease transmission model, the underlying social contact network, and perfect testing. In real-world scenarios, perfect testing is unlikely to be achieved and quarantine compliance may vary. There are considerable uncertainties around the transmission of SARS-CoV-2, especially regarding age-related factors, the asymptomatic cases, and their transmission. We also do not include the possible reintroduction of SARS-CoV-2 in the population from externally infected individuals in the simulation. These may result in fewer simulated infections than in the real scenario. Nonetheless, our findings contribute to the understanding of the potential effectiveness of adaptive group testing strategies in controlling infectious diseases and offer insights that can inform policy and public health decision-making.

AGT takes advantage of social contact network partitioning to improve the efficiency of group testing. The partitioning process divides the population into subgroups based on social contact interactions, grouping individuals who have social contacts together within the same subgroup. These subgroups, defined by the social contact network, remain consistent throughout subsequent testing rounds, ensuring stability and continuity in the testing strategy. This fixed grouping approach enables easier implementation and meaningful comparisons of results across multiple testing rounds. It also supports the traceability of infection status within each subgroup over time, as individuals within the same subgroup share similar social contacts and interactions. While the fixed grouping approach provides valuable insights into infection spread within specific social contact networks, it is important to acknowledge that social networks are dynamic and subject to changes. Different optimal groupings may emerge at different time instances as social contact patterns evolve. Recognizing the evolving nature of social contacts, future research could explore group testing under dynamically changing groupings. This could involve incorporating time-series mobility networks or other time-dependent factors to account for the changing dynamics of social contacts and its impact on the effectiveness of group testing strategies. Evaluating AGT on mixed group sizes in real-world settings and considering factors such as demographic information, geographical distribution, and contact patterns would further enhance its effectiveness and optimize testing outcomes. These investigations would provide a deeper understanding of how adaptive groupings can enhance testing strategies in response to the evolving nature of social contact patterns.

Inadequate testing of exposed individuals may lead to further spread of the infection among susceptible individuals due to undetected infected individuals. Our study highlights the importance of considering the dynamic changes in virus transmission within the social contact network. While a higher testing capacity can result in a faster containment of the spread of the virus, our simulations indicate that a testing capacity of 15% of the total population is sufficient to achieve acceptable results. This allows for a more efficient allocation of resources, enabling the use of these resources on a larger scale.

Additionally, for every scenario and method, an optimal group size can be determined. However, the group size is also constrained by the practical limitations of the testing methodology, such as dilution effects and sensitivity considerations. This can prevent choosing the optimal group size for a low prevalence scenario. When the underlying social contact network is unknown, smaller group sizes may work better than larger ones as it may reduce the uncertainty associated with identifying the best group size. To determine the optimal group size in such cases, it is important to take into account a range of group sizes in simulations, evaluate the performance of the group testing strategy for each group size, and further identify the group size that results in the most efficient use of resources.

Note that the advantage of AGT in reducing outbreak size is not significant when the total population gradually increases. This may be because the shortest path between infected individuals becomes longer. Each infected individual gains a larger transmittable space when the total population increases. At this point, without any testing, the initially infected individuals may be able to infect all neighbors to reach the theoretical upper limit number of secondary transmissions. However, no matter which group testing strategy is applied, the initially infected individuals can always be detected after testing begins in a few days (time points). Due to the slight difference in detection time, it is difficult to have a small regional outbreak caused by an undetected infected individual. AGT enables early detection of infected individuals by tracking their infection trajectory, thereby containing the spread of the disease more quickly and protecting more susceptible individuals. An earlier end to transmission also saves more resources on follow-up testing.

The smaller advantage of AGT over RGT in real social networks compared to synthetic networks may be due to the higher density of the real social networks used in this study, allowing for faster and wider virus spread. However, it is important to note that in real-world scenarios, the duration of contact plays a crucial role in disease transmission, emphasizing the significance of contact intensity rather than simply the presence of contact. Simulating the disease on high-density networks may result in an overestimation of the spread. Additionally, RGT, which adopts a random average sampling approach, can achieve maximum coverage with limited tests, leading to higher hit rates, particularly in high-density scenarios. To enhance the effectiveness of AGT in real-world high-density networks, it is essential to consider contact duration and explore alternative sampling strategies tailored to these specific network characteristics.

In addition to the scenario studied in this research, our group testing strategy also has the potential to be applied to other infectious diseases, such as tuberculosis, HIV, and influenza, as the underlying principle of early identification of infected individuals and reduction of the burden on the public health system remains consistent across different diseases with similar transmission patterns. However, it is essential to note that the specific implementation of the strategy may need to be tailored to the characteristics of each disease, such as the level of asymptomatic transmission and the sensitivity of the testing method. Besides, the assumptions of the transmission model and underlying social contact network may also need to be reevaluated for each specific disease. Future research should focus on tailoring and validating the AGT strategy for specific infectious diseases to maximize its potential impact in various real-world scenarios.

Moreover, extending the group testing strategy to more extensive and complex social contact networks could potentially improve its effectiveness. The performance of AGT is partially dependent on the underlying structure of the social contact network. As the size of the social contact network increases, the probability of infected individuals being connected to each other through multiple pathways increases, and the underlying correlation within each testing group also grows, which could make it easier to identify infected individuals through AGT. A deeper understanding of the impact of graph structure on the effectiveness of testing methods is crucial. To shed light on this, we conducted several simulations on different synthetic network models, aiming to explain the performance of AGT under various network structures. These simulations provide insights into the behavior of AGT when only partial information of the real underlying contact network is available, further emphasizing the need for future studies to explore the effectiveness of AGT in real-world scenarios with incomplete graph structure information. In addition, it is important to note that as the size of the social contact network increases, the complexity of the network also increases, which may make it more challenging to identify the optimal group size and testing strategy, as well as to collect and analyze the data on such large social contact networks. Further research is necessary to evaluate the generalizability of the group testing strategy in more extensive social contact networks and to determine the specific parameters that need to be adjusted to optimize its performance, taking into account the challenges of scaling up the method.

Implementing AGT in real-world settings can be challenging due to various factors. One key challenge is the cost associated with group testing, including expenses for testing kits, personnel, and infrastructure. Additionally, the coordination of large-scale testing efforts, ensuring timely delivery of test results, and managing the logistics of testing individuals within each group require collaboration between healthcare organizations, government agencies, and community stakeholders. However, these challenges can be addressed through strategic resource allocation, adopting cost-effective testing approaches, and efficient logistical planning. Technological advancements, such as automation and high-throughput testing methods, can streamline testing processes and reduce costs. Public awareness campaigns can also play a role in encouraging participation and facilitating the smooth implementation of AGT strategies in real-world settings.

## Conclusion

The standard two-stage pooling approach usually pools individuals randomly. As the sample correlation increases, the efficiency of pooling without prior information drops rapidly. In this work, we propose an adaptive group testing strategy based on the social contact network with fixed, limited testing capacity at each time instance. Individuals sharing similar social contacts with neighbors are pooled together using our graph-partition based strategy. To facilitate the simulation, we develop an enhanced compartment model that captures the SARS-CoV-2 spreading characteristics in the COVID-19 pandemic based on the classic SEIR model. We evaluate AGT with other group testing methods and individual testing methods using simulated epidemics on both real and synthetic social contact networks. The results of this study show that our adaptive group testing strategy can save up to 88.41% tests on the real contact networks and 90.56% on synthetic networks. This can help reduce the time and resources needed to test many people. Moreover, our approach helps lower the outbreak size up to 84.31% and postpones the arrival of the peak, which will assist in relieving the pressure on public health resources during the outbreak.

**Figure 2:**
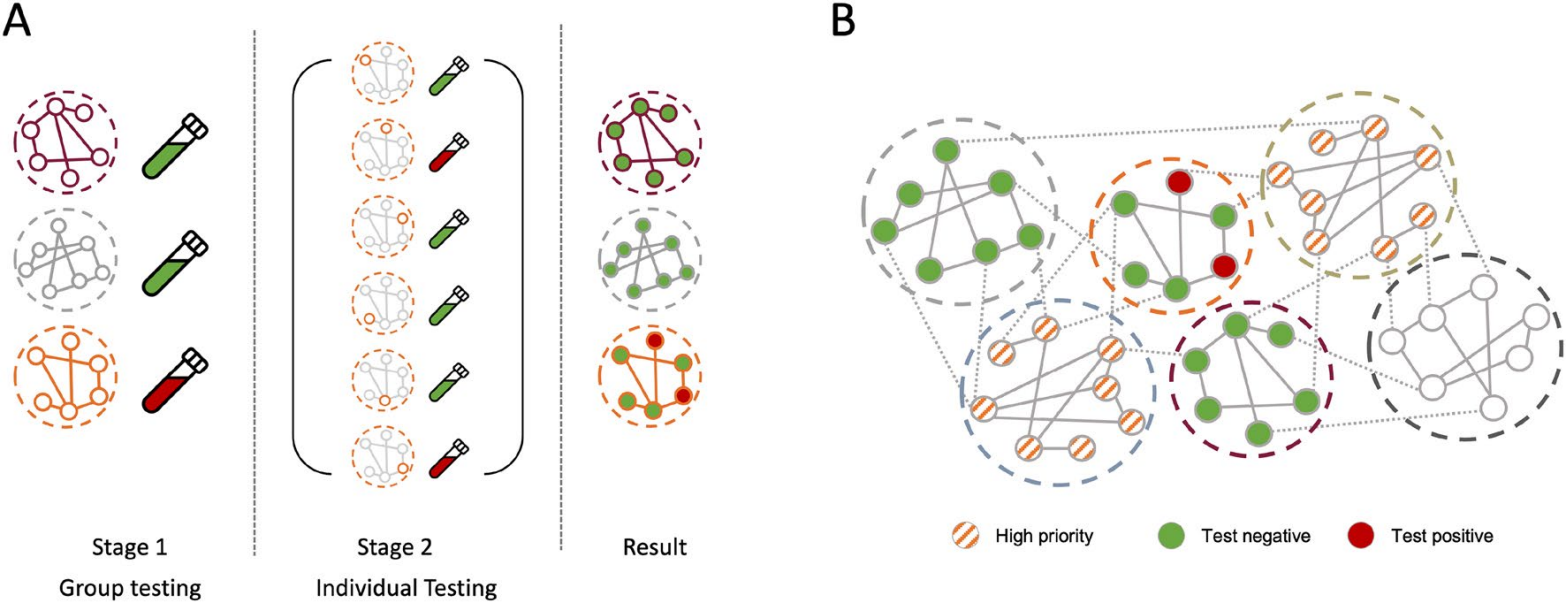
(**A**) The two-stage testing design that is adapted from Dorfman’s method^8^. (**B**) Adjusting the testing order according to previous testing results, the point prevalence, and the topological connectivity of each group.

**Figure 3:**
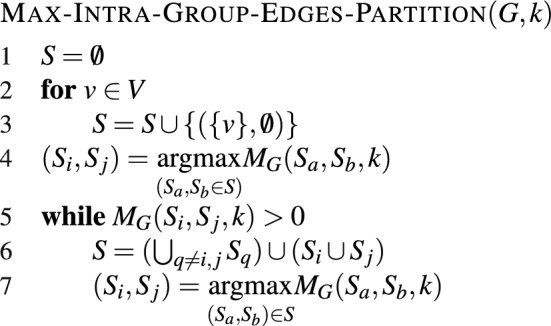
Algorithm for dividing the vertices of the given undirected graph into k sets while maximizing the number of edges within each set.

**Figure 4:**
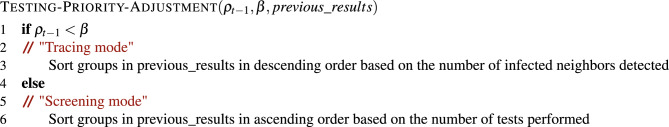
Algorithm for adjusting the testing priority of groups adaptively based on the point prevalence and previous test results.

**Figure 5:**
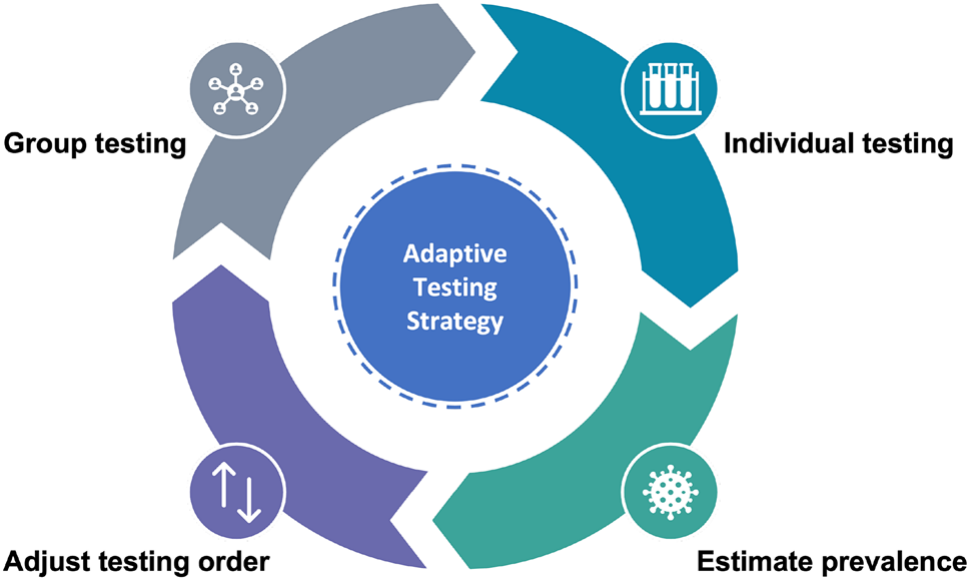
The adaptive testing strategy, given as a processing cycle over time.

**Figure 6:**
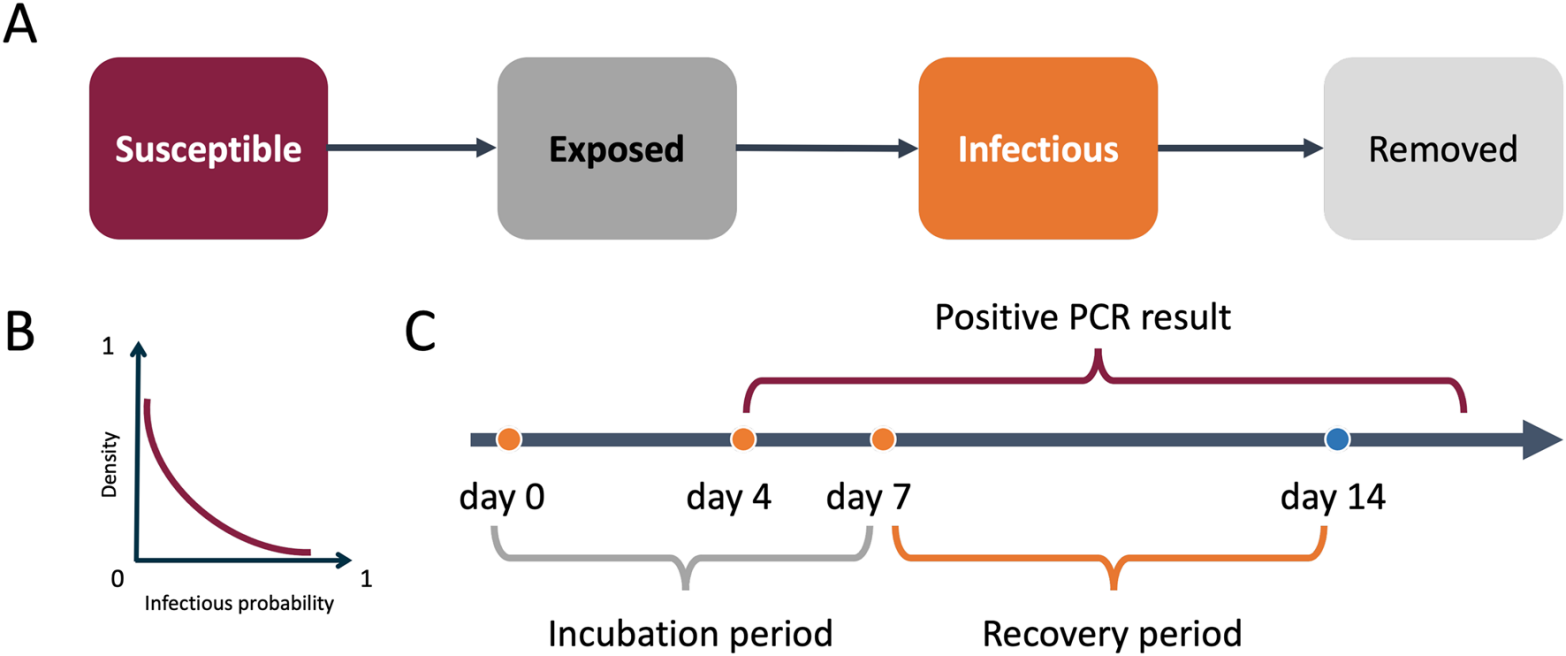
(**A**) Agent based Infectious disease transmission model. (**B**) The exponential distribution for modeling the infectious heterogeneity. (**C**) Distinct periods of the enhanced SEIR model.

**Figure 7:**
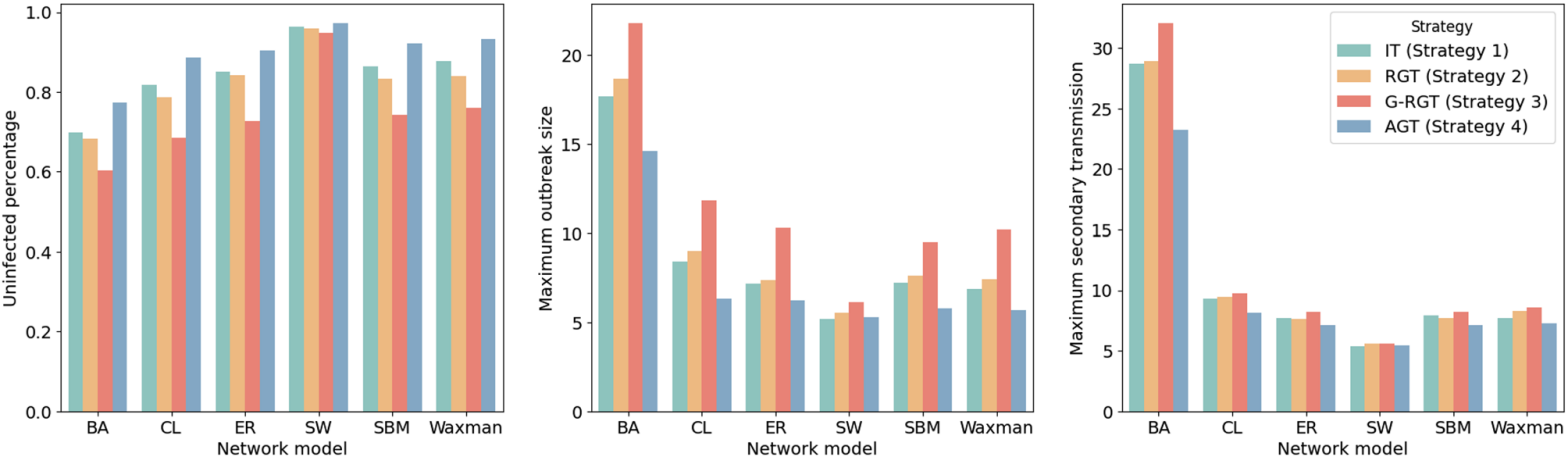
Simulation results of our strategies and baselines using six network generation models. The total population is 1000. AGT outperforms all competing strategies in protecting more susceptible individuals, reducing outbreak size, and reducing secondary transmissions.

**Figure 8:**
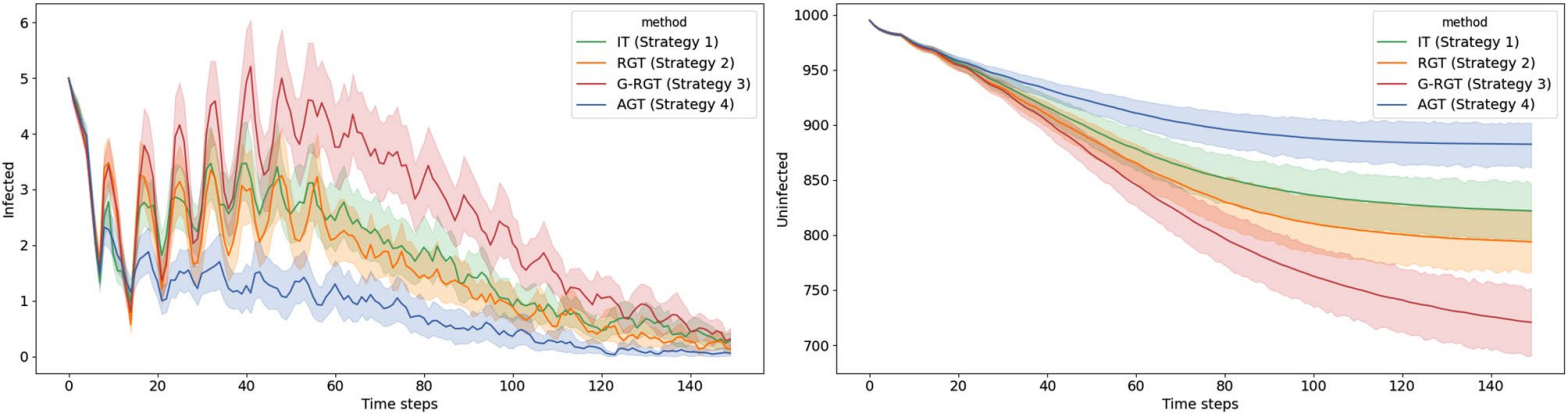
Simulation results of our strategies and baselines on the Chung-Lu network model (CL). The total population is 1000. The left figure reports the number of infected individuals per day. The right figure shows the number of uninfected individuals per day. The shaded region around each line is the corresponding 95% confidence interval. AGT steadily reduces the number of infections, decreases the outbreak size, and protects more susceptible individuals.

**Figure 9:**
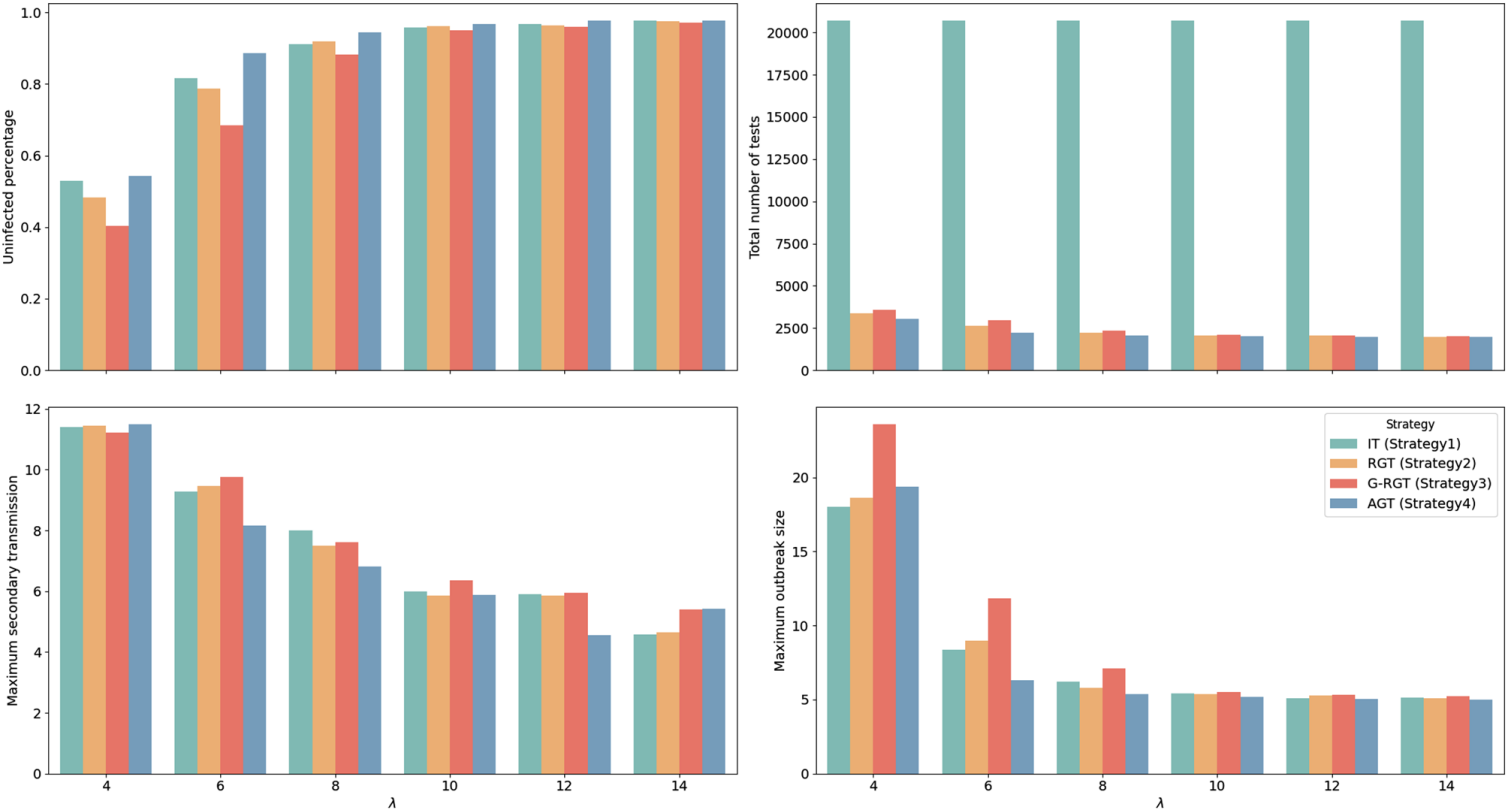
Effectiveness of AGT and baselines on the CL network with varying transmission settings. AGT demonstrates a higher percentage of uninfected individuals and a lower total number of tests, indicating its effectiveness in minimizing infections while optimizing testing resources. The performance in controlling secondary infections remains stable, and the maximum outbreak size can be effectively controlled to the initial outbreak size (5) within a $\lambda$ range of 6–14. Additionally, AGT exhibits superior performance at higher transmission rates, achieving better control of outbreaks with lower resource consumption.

**Figure 10:**
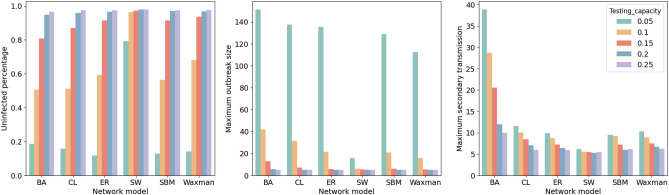
Testing performance for varying testing capacities on different network models. The larger test capacity provides better performance in all measurements. The differences are not significant after it exceeds 15%.

**Figure 11:**
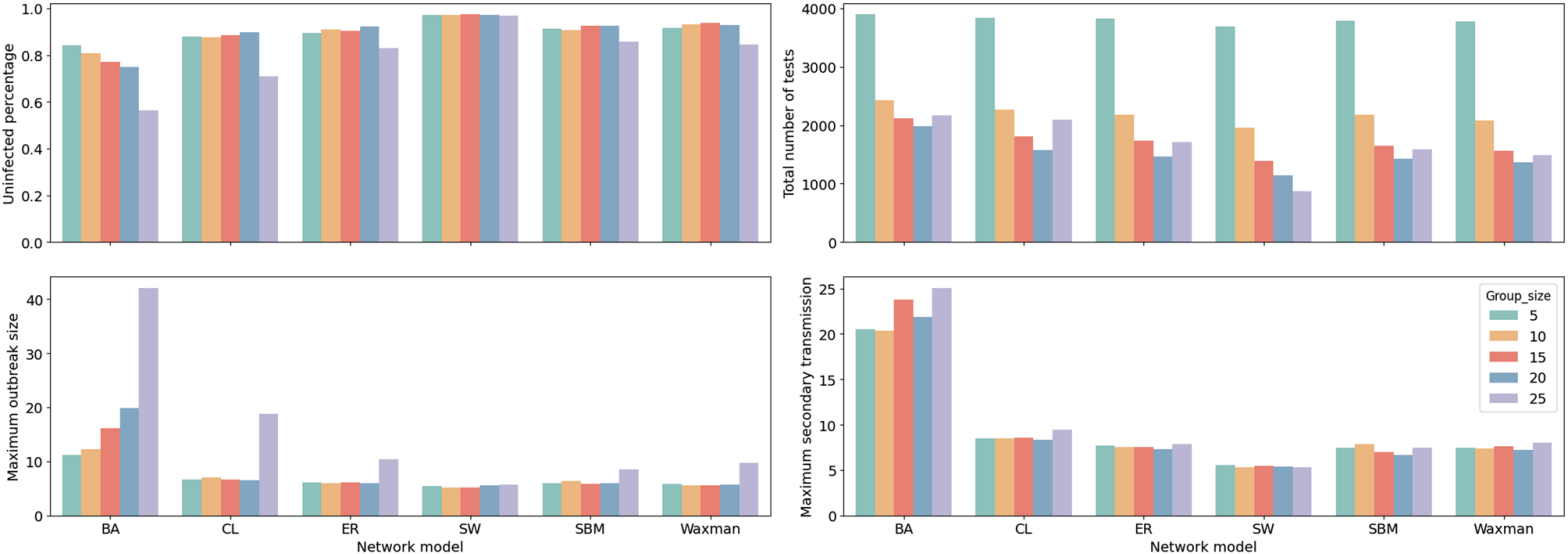
Testing performance for different network models based on varying group sizes. The optimal group size is not consistent for different network structures. Smaller groups perform better on lowering the outbreak size than larger ones, while larger group size can save more testing resources.

**Figure 12:**
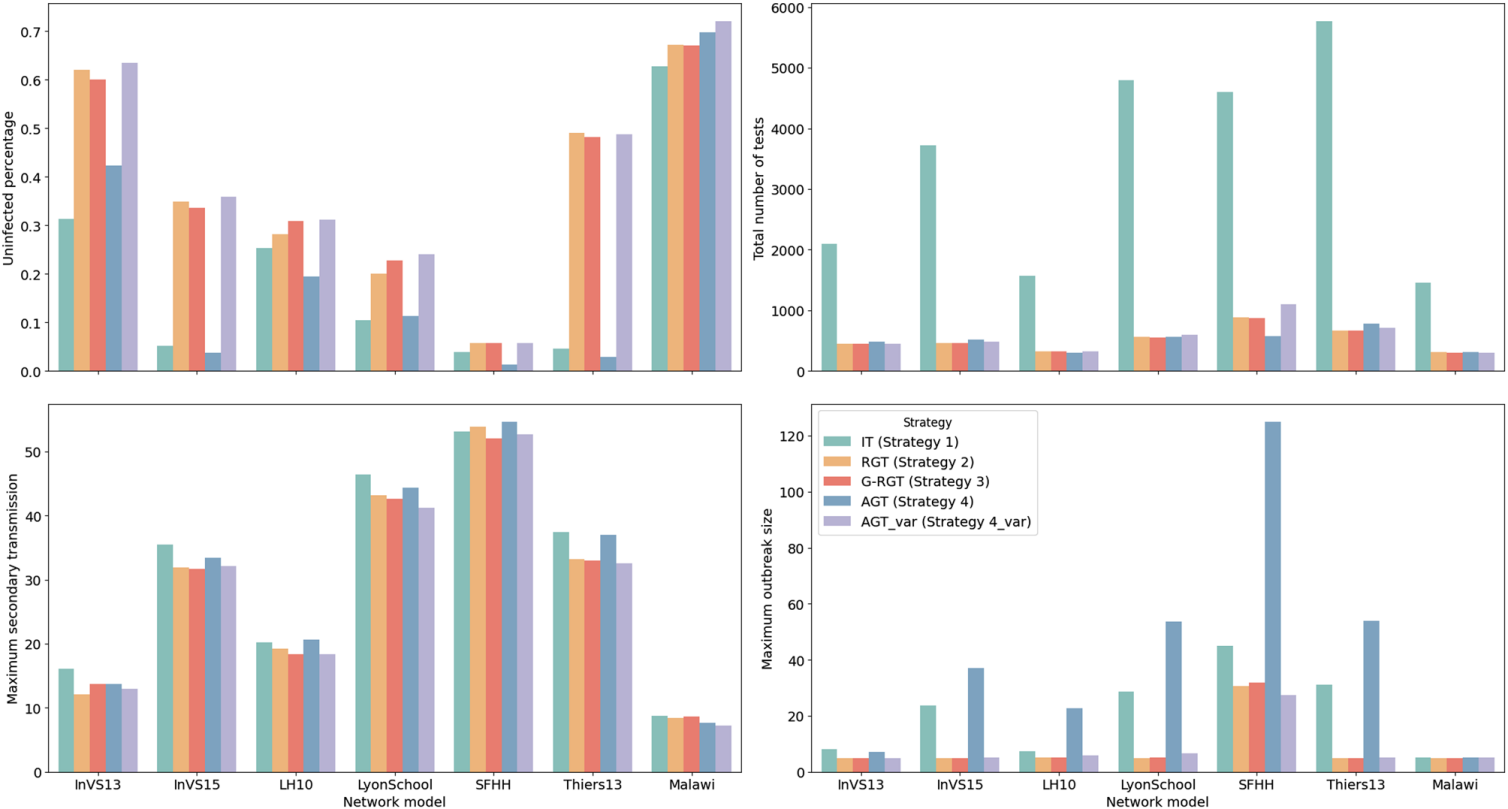
Simulation results of our strategies and baselines using seven real contact networks. Group testing strategies (Strategy 2, 3, 4, and its variation) outperform Individual Testing (IT) (Strategy 1), saving 76.08–88.41% of testing resources. However, the difference among different group testing strategies is insignificant. Notably, the variant designed for high-density networks shows significant reduction only on the SFHH network.

**Figure 13:**
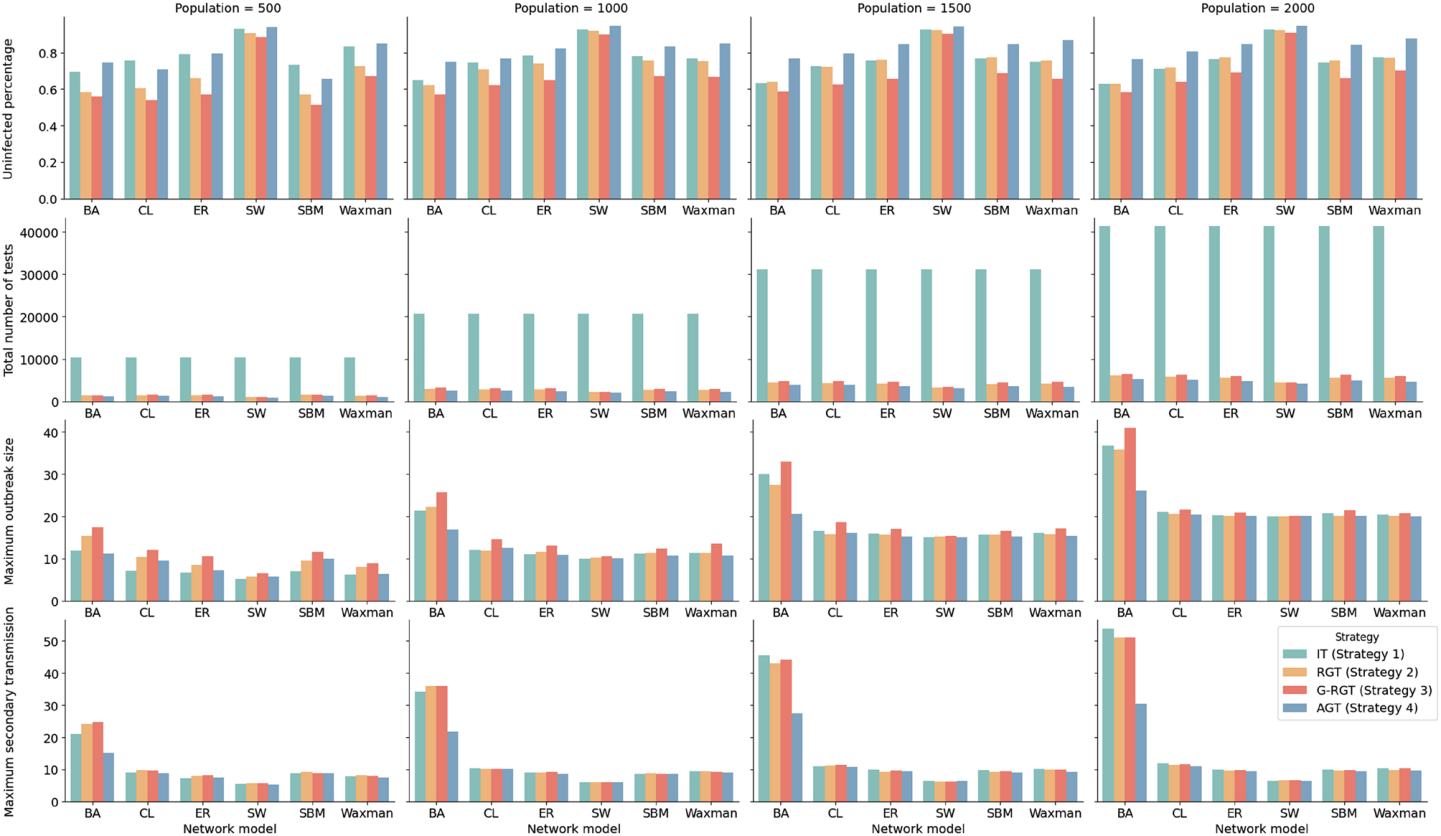
Simulation results of our strategies and baselines based on varying population scales. The initial outbreak size is set at 1% of each population, and the test capacity is set at 15% of the corresponding population. Our proposed strategy outperforms the other strategies in terms of reducing the total number of tests and outbreak size.

**Table 1:** Nomenclature of notations and parameters.

Notations	Description
*G*	Undirected social contact network
$G_i$	*i*th subgraph of *G*
*V*	Vertex set of *G*
*E*	Edge set of *G*
*m*	Number of subgraphs
$M_G(G_i, G_j, k)$	Partition score function for calculating the maximum number of edges in the combined subgraphs without exceeding size *k*
*S*	Susceptible agent status
*E*	Exposed agent status
*I*	Infectious agent status
*R*	Removed/recovered agent status
$V_i$	*i*th testing group
$X_{i,t}$	Group test result on testing group $V_i$ at time *t*
$Q_{i,t}$	The number of tests performed on testing group $V_i$ at time *t*
$\rho _t$	Point prevalence at time *t*
$\beta$	Strategy mode switch threshold
$z_t$	Number of group tests at time *t*
*y*	Number of infected individuals detected by our testing strategy
*N*	Number of total population
*k*	Upper size bound of the testing group
*m*	Number of testing groups
*T*	Testing period
*B*	Testing capacity

**Table 2:** Synthetic social contact networks used for the simulation.

Generation model	*N*	$\left\langle k \right\rangle$	*d*	Ref.
Barabási–Albert model (BA)	1000	5.96	5	^34^
Chung–Lu model	1000	5.82	7	^35^
Erdos–Renyi model (ER)	1000	5.98	7	^36^
Stochastic Block model (SBM)	1000	6.00	10	^37^
Watts–Strogatz Small-world model (SW)	1000	6.01	7	^38^
Waxman’s model (Waxman)	1000	6.24	11	^39^

**Table 3:** Real social contact networks used for the simulation.

Data Set	Location	Year	*N*	*T*	$\left\langle k \right\rangle$	*d*	Ref.
InVS13	Fr. Health Obs.	2013	92	2 weeks	13.15	4	^28^
InVS15	Fr. Health Obs.	2015	232	2 weeks	38.38	5	^28^
LH10	Hospital	2010	81	3 days	22.02	3	^29^
LyonSchool	Primary school	2009	242	2 days	48.43	3	^30^
SFHH	Conference	2009	403	2 days	47.47	4	^31^
Thiers13	High school	2013	326	1 week	34.71	4	^32^
Malawi	Village	2021	86	26 days	8.07	5	^33^

**Table 4:** Parameters of enhanced SEIR model simulation.

Parameter	Description	Value
$N_0$	Initial outbreak size	5
$\lambda$	Parameter of the infectious distribution $X\sim \textrm{Exp}(\lambda )$	6
$\alpha$	Incubation period	7
$\mu$	Mean value of the recovery period	7
$\sigma$	Standard deviation of the recovery period	4

**Table 5:** Comparison of testing strategies simulated on six synthetic contact networks with 1000 population.

	Strategy	BA	CL	ER	SW	SBM	Waxman
Uninfected	IT	697.96	816.78	851.84	963.87	865.17	876.47
	RGT	683.09	786.74	842.12	959.48	833.87	840.38
	G-RGT	603.76	685.04	727.41	947.67	741.39	760.88
	AGT	**773.61**	**886.63**	**903.84**	**973.1**	**921.43**	**932.92**
Maximum outbreak size	IT	17.65	8.39	7.15	**5.2**	7.22	6.87
	RGT	18.67	8.98	7.35	5.51	7.6	7.42
	G-RGT	21.79	11.83	10.31	6.11	9.47	10.17
	AGT	**14.58**	**6.31**	**6.24**	5.29	**5.77**	**5.68**
Maximum secondary transmission	IT	28.71	9.29	7.71	**5.38**	7.91	7.69
	RGT	28.92	9.46	7.62	5.59	7.74	8.3
	G-RGT	32.09	9.77	8.25	5.61	8.2	8.6
	AGT	**23.27**	**8.17**	**7.16**	5.48	**7.16**	**7.26**
Total tests	IT	20700	20700	20700	20700	20700	20700
	RGT	2874.67	2626.04	2457.96	2059.86	2492.2	2468.61
	G-RGT	3138.59	2976.14	2864.47	2066.1	2818.68	2714.02
	AGT	**2504.26**	**2241.37**	**2195.71**	**1953.8**	**2139.22**	**2085.01**
Found positives	IT	247.69	149.68	121.23	28.89	109.78	100.35
	RGT	294.37	196.86	144.82	35.59	152.49	146.33
	G-RGT	**365.97**	**288.99**	**247.65**	**46.01**	**235.43**	**218.4**
	AGT	195.27	102.45	86.77	23.09	70.89	60.84

Significant values are in [bold].

**Table 6:** Comparison of testing strategies simulated on seven real contact networks.

	Strategy	InVS13	InVS15	LH10	LyonSchool	SFHH	Thiers13	Malawi
Uninfected	IT	39.18	11.69	23.75	29.87	15.71	16.16	53.9
	RGT	77.45	77.7	26.45	57.68	**23.39**	**169.09**	57.7
	G-RGT	75.09	74.9	29	65.32	22.94	166.31	57.58
	AGT	52.87	8.52	18.31	32.54	5.48	10.07	59.92
	AGT_var	**79.22**	**79.98**	**29.28**	**68.84**	23.17	168.25	**61.99**
Maximum outbreak size	IT	8	23.75	7.46	28.65	45	31.2	5.15
	RGT	**5**	**5**	**5.14**	**5**	30.7	**5**	**5**
	G-RGT	**5**	**5**	5.19	5.07	31.97	**5**	**5**
	AGT	7.1	37.02	22.62	53.7	124.97	53.9	5.25
	AGT_var	**5**	5.06	5.75	6.5	**27.41**	5.14	5.01
Maximum secondary transmission	IT	16.15	35.53	20.18	46.43	53.14	37.39	8.75
	RGT	**12.1**	31.92	19.21	43.13	53.95	33.21	8.44
	G-RGT	13.69	**31.75**	**18.43**	42.67	**52.07**	32.97	8.59
	AGT	13.74	33.46	20.61	44.41	54.66	37.01	7.71
	AGT_var	12.96	32.17	**18.43**	**41.28**	52.7	**32.56**	**7.21**
Total tests	**IT**	2098	3722	1574	4795	4602	5772	1453
	RGT	**445.66**	**455.94**	324.68	558.11	880.43	669.1	306.67
	G-RGT	446.54	456.75	320.77	**556.28**	875.95	**670.01**	305.89
	AGT	479.07	515.19	**304.38**	561.86	**579.94**	775.54	306.89
	AGT_var	452.95	481.57	328.78	599.89	1100.63	710.53	**303.91**
Found positives	IT	**63.59**	**156.63**	54.39	191.12	311.73	**238.19**	24.49
	RGT	45.41	143.46	**65.18**	**226.9**	367.51	174.03	26.16
	G-RGT	47.99	146.34	62.37	219.62	**367.59**	176.89	**26.3**
	AGT	61.61	145.03	36.61	154.34	166.03	220.84	21.95
	AGT_var	43.82	140.87	62	215.26	366.9	174.56	21.88

Significant values are in [bold].

## Data Availability

All the Python scripts that were used in this research are available in the GitHub repository at https://github.com/EveZhang19/CompEpi_AGT.
